# Energy-efficient CO_2_ hydrogenation with fast response using photoexcitation of CO_2_ adsorbed on metal catalysts

**DOI:** 10.1038/s41467-018-05542-5

**Published:** 2018-08-02

**Authors:** Chanyeon Kim, Seokwon Hyeon, Jonghyeok Lee, Whi Dong Kim, Doh C. Lee, Jihan Kim, Hyunjoo Lee

**Affiliations:** Department of Chemical and Biomolecular Engineering, Korea Advanced Institute of Science and Technology, Daejeon, 34141 South Korea

## Abstract

Many heterogeneous catalytic reactions occur at high temperatures, which may cause large energy costs, poor safety, and thermal degradation of catalysts. Here, we propose a light-assisted surface reaction, which catalyze the surface reaction using both light and heat as an energy source. Conventional metal catalysts such as ruthenium, rhodium, platinum, nickel, and copper were tested for CO_2_ hydrogenation, and ruthenium showed the most distinct change upon light irradiation. CO_2_ was strongly adsorbed onto ruthenium surface, forming hybrid orbitals. The band gap energy was reduced significantly upon hybridization, enhancing CO_2_ dissociation. The light-assisted CO_2_ hydrogenation used only 37% of the total energy with which the CO_2_ hydrogenation occurred using only thermal energy. The CO_2_ conversion could be turned on and off completely with a response time of only 3 min, whereas conventional thermal reaction required hours. These unique features can be potentially used for on-demand fuel production with minimal energy input.

## Abstract (web summary)

While many heterogeneous chemical transformations require high temperatures, such conditions are costly and corrosive to the catalysts. Here, authors enhance CO_2_ hydrogenation over metal nanoparticles by light irradiation via an unusual mechanism and reduce the reaction’s energetic demands.

## Introduction

Metal nanoparticles loaded onto supports have been typically used as heterogeneous catalysts for surface reactions such as hydrogenation, oxidation, reforming, and coupling reactions^1–5^. Surface reactions often require high temperatures to overcome the activation energy barrier. Operating the reactor at a high temperature may demand excessive energy and stringent safety controls. Recently, light-assisted surface reactions have been reported^6–11^. Using heat and light together as two different energy sources might minimize the overall energy usage and provide unique features, which cannot be provided by conventional thermal catalytic reactions.

Enhancing the catalytic activity under light irradiation has been reported for heterogeneous reactions including ethylene epoxidation on Ag nanocubes, reverse water–gas shift reaction on Al@Cu_2_O, ethanol dehydrogenation on Ag–Ni nanosnowmans^6–8^. Light is absorbed on the catalyst and then transferred to reactant molecules on the surface by heat or hot electrons. The photothermal effect, in which absorbed light energy is relaxed as heat, would occur only with a very strong light that is orders of magnitude higher than the intensity of sunlight^12–15^.

Hot electrons are generated on metal nanoparticles by an intraband or interband transition upon light absorption. Localized surface plasmon resonance (LSPR) is a typical intraband transition that can be derived from metals with high electron mobility in sp-band. Group IB metals such as Au, Ag, and Cu are representative plasmonic metals and other transition metals also can have LSPR occasionally^8,9,16–18^. Non-plasmonic metal nanoparticles can produce hot electrons by interband transition, in which electrons are excited from d-band to unoccupied conduction band^19–22^. However, light absorption by the interband transition is usually weak, and it is effective only under UV light. When the hot electrons contribute to the surface reaction, the catalytic activity for various wavelengths of incident light typically follows the trend of their light absorption spectra. With an increase in the light absorption, more hot electrons are produced, thereby enhancing the catalytic reaction further.

However, this analogy might not be applicable when light is absorbed by direct excitation of the electrons located at the hybrid orbitals of a reactant adsorbed on a metal surface. When a reactant molecule is strongly adsorbed on a metal, the energy levels of electronic orbitals are rearranged. If the energy gap between the highest occupied molecular orbital (HOMO) and the lowest unoccupied molecular orbital (LUMO) is reduced, then the incident light can excite the electrons on the hybrid orbitals promoting the surface reaction^10,23–25^. Christopher et al. reported that CO oxidation in a H_2_-rich stream can be promoted by direct photoexcitation of adsorbate (CO)–metal (Pt) bonds^10^. Zhu et al. also observed selective oxidation of aliphatic alcohols on Pt or Pd nanoparticles under visible light irradiation^11^.

Here, we show that CO_2_ hydrogenation is enhanced on the Ru surface under light irradiation. The effects of the metal type, Ru domain size, and Ru oxidation state are evaluated. The DFT calculation is performed to investigate the changes in the energy gap between the HOMO and LUMO levels for various metals. Light irradiation reduces the reaction temperature significantly minimizing the total energy usage for CO_2_ hydrogenation. Additionally, the reaction can be turned on or off in a moment, unlike the conventional thermal reaction.

## Results

### Enhancement in activity upon light irradiation

**Fig. 1:**
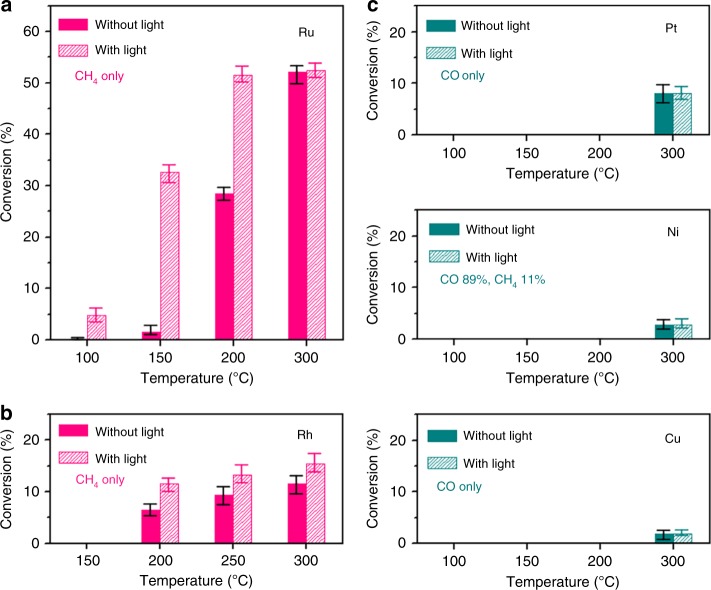
Light enhancement on metal catalysts. CO_2_ conversion for CO_2_ hydrogenation on **a** Ru, **b** Rh, and **c** Pt, Ni, and Cu catalysts deposited onto silica with and without light irradiation. 0.5 vol% CO_2_/N_2_ (50 sccm) and H_2_ (1.5 sccm) was fed into the reactor through a static mixer with or without light irradiation. A Xe lamp with light intensity of 35 mW cm^−2^ was used with a water-circulating filter to exclude any thermal effect from the light source. The error bar indicates the deviation among three independent measurements

Various metals (i.e., Ru, Rh, Pt, Ni, and Cu) were deposited on silica supports using a wet impregnation method. The TEM images of the catalysts were shown in Supplementary Fig. 1. The size of the metal nanoparticles was controlled to be between 5 and 7 nm by changing the weight percentages of the metals as shown in Supplementary Table 1. The powder catalysts containing 10 mg of metal were loaded into the custom-made photoreactor. A schematic of the reactor set-up and its photograph are shown in Supplementary Fig. 2. CO_2_ hydrogenation was performed by flowing CO_2_ and H_2_ together after reducing the catalysts at 300 °C for 3 h. Figure 1 shows the CO_2_ conversion data of various metals with and without light irradiation (Supplementary Table 2 for the full data). Ru and Rh produced CH_4_ only, whereas Pt and Cu produced CO only. Ni produced 89% CO and 11% CH_4_. The CO_2_ conversion increased upon light irradiation for Ru and Rh, while the conversion did not change for Pt, Ni, and Cu. Especially, the Ru catalyst showed remarkable enhancements. At 150 °C, the CO_2_ conversion was 1.6% without light, but it increased to 32.6% with light. This conversion was obtained at 230 °C without light. By simply shining light, the reaction temperature for CO_2_ hydrogenation was lowered significantly. When a higher concentration of CO_2_ was used with CO_2_/N_2_/H_2_ flow rates of 5/30/15 sccm, the CO_2_ conversion was also significantly enhanced from 1.1 to 10.9% at 150 °C upon light irradiation as shown in Supplementary Fig. 3. Supplementary Table 3 shows the comparison of the production rate with the literature values reported for gas-phase CO_2_ hydrogenation performed under light irradiation^13,26–29^.

**Fig. 2:**
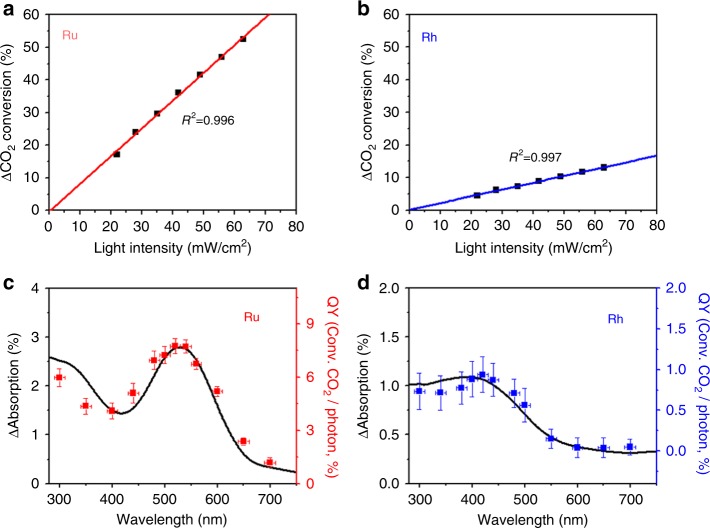
Dependence on light intensity and light wavelength. **a**, **b** CO_2_ conversion for CO_2_ hydrogenation on Ru/SiO_2_ and Rh/SiO_2_ when the light intensity was varied. To clarify the relation between the light intensity and CO_2_ conversion enhanced by the light, thermal CO_2_ conversion without light was subtracted from light-assisted CO_2_ conversion (ΔCO_2_ conversion). Light intensity after passing the sapphire window was measured by a spectroradiometer. **c**, **d** In-situ UV–Vis spectroscopy in CO_2_ flow (black lines) and quantum yield for wavelength-dependent measurement of CO_2_ hydrogenation on Ru/SiO_2_ or Rh/SiO_2_ (red or blue dots). Error bars in *x*-direction indicates full-width at half maximum intensity of monochromatic light. Error bars in *y*-direction indicates a deviation among three different measurements. The in-situ UV–Vis spectroscopy and CO_2_ conversion was measured at 150 °C for Ru/SiO_2_ and 200 °C for Rh/SiO_2_

The light intensity during the CO_2_ hydrogenation was 35 mW cm^−2^, which is too weak to induce any photothermal effect. The photothermal effect has been observed in a light intensity range of 1–10^6^ W cm^−2^, regardless of the metal type^12–15,30^. The intensity of sunlight is ~100 mW cm^-2^. A linear relation between the photocatalytic activity and light intensity has been well known as a fingerprint for hot electron-driven chemical reactions^7,8,31,32^. Figure 2a and b shows that the CO_2_ conversion increased linearly as the light intensity increased on the Ru or Rh catalysts. Clearly, the light-enhanced CO_2_ hydrogenation results from the hot electrons.

The silica support is photochemically inert; thus, Ru or Rh should provide hot electrons. The extent of the surface oxide was changed to observe the effect of the surface oxide for the light enhancement. While the as-made Ru/SiO_2_ catalyst with a Ru size of 5.6 nm has 89% of metallic Ru and 11% of Ru oxide at the surface, the percentages of Ru oxide increased to 44 and 61% after annealing the catalyst at 500 °C in air for 1 and 60 min, respectively (Supplementary Fig. 4). Supplementary Fig. 5 shows the CO_2_ conversion data for these samples having different amounts of Ru oxide with and without light irradiation. The enhancement from the light decreased drastically as the amount of Ru oxide increased, and the sample annealed for 60 min showed little enhancement upon light irradiation. This indicates that metallic Ru is responsible for the enhancement by light.

The effect of the Ru nanoparticle size was estimated. Different sizes (2.6, 5.6, and 17.1 nm) of Ru nanoparticles were obtained by changing the weight percentages of Ru on the Ru/SiO_2_ catalyst, and their TEM images are shown in Supplementary Fig. 6. The nanoparticle sizes were checked using both powder XRD and the H_2_ uptakes as shown in Supplementary Table 4. The fraction of metallic Ru on the surface was the same as ~90%. Supplementary Fig. 7 shows that the enhancement by light was much smaller in the 2.6 nm Ru nanoparticles, while both the 5.6 and 17.1 nm Ru nanoparticles had a distinct enhancement. It was reported that larger Ru nanoparticles (e.g., ≥5 nm) exhibit a higher CO_2_ conversion than smaller ones for conventional CO_2_ hydrogenation because CO_2_ dissociation requires higher activation barrier on smaller Ru nanoparticles^33–35^. Thus, we hypothesized that the enhancement by light may be related to the interaction between the Ru surface and the CO_2_ molecule.

Hot electrons can be produced on a metal surface by an intraband or interband transition^8,9,16–22^. Among Ru, Rh, Pt, Ni, and Cu, only Cu can have an intraband transition; however, the Cu catalyst did not show any enhancement for CO_2_ hydrogenation under light. If hot electrons produced by the interband transition promote CO_2_ hydrogenation, all the metals, not just Ru or Rh, should show an enhancement. Additionally, the interband transition is usually observed in the ultraviolet (UV) range, and the enhancement by the interband transition decreases abruptly as the wavelength of the incident light increases^20,22^. In-situ UV–Vis spectroscopy was performed in CO_2_ flow as shown in Fig. 2c, d (black lines). While UV-DRS data (Supplementary Fig. 8) obtained without CO_2_ showed no peak, the peaks were observed at 528 nm for Ru/SiO_2_ and 410 nm for Rh/SiO_2_. When the content of Ru/SiO_2_ catalyst in KBr mixture increased, the absorbance also increased without the change in peak position as shown in Supplementary Fig. 9. Interestingly, these spectra from in-situ UV–Vis measurements (black lines) agree well with the photoaction spectra, which are the plots of quantum yield (QY) vs. wavelength (red or blue dots in Fig. 2c, d). The QY was estimated from the CO_2_ hydrogenation reaction data at each wavelength using a monochromator. Briefly, QY was calculated by dividing the number of converted CO_2_ molecules with the number of incident photons at each wavelength (Supplementary Fig. 10). The detailed procedure is explained in Methods. The photoaction spectra also presented peaks at 520 nm for Ru/SiO_2_ and 420 nm for Rh/SiO_2_. This result indicates that the hot electrons originate from light absorption by CO_2_ adsorbed on the metallic Ru or Rh surface and they promote the CO_2_ hydrogenation.

### Mechanism study

**Fig. 3:**
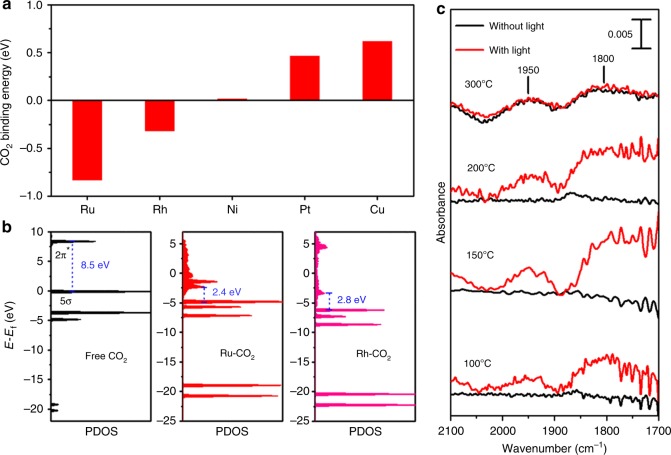
Binding of CO_2_ on metal surfaces. **a** CO_2_ binding energy and **b** HOMO-LUMO gap of free CO_2_, adsorbed CO_2_ on the Ru surface, or adsorbed CO_2_ on the Rh surface, obtained using DFT calculations. **c** DRIFT spectra for CO_2_ adsorption on Ru/SiO_2_ with or without light irradiation. 50 sccm of 0.5% CO_2_/N_2_ was flowed for 10 min; the cell was purged with 60 sccm of N_2_ for 15 min to desorb the weakly adsorbed CO_2_, and the IR spectra were obtained

The CO_2_ binding energy on various metal surfaces was estimated using density functional theory (DFT) calculations, as shown in Fig. 3a. (111) surface was used for the face-centered cubic metals of Rh, Pt, Ni, and Cu, while (0001) surface was used for the hexagonal close packed metal of Ru. Various CO_2_ positions were tested to obtain the minimum energy configuration as shown in Supplementary Fig. 11. Figure 3a shows the values of the lowest CO_2_ binding energy. Supplementary Fig. 12 shows the state of CO_2_ with the lowest energy configurations. Ru and Rh have a negative binding energy, while Pt, Ni, Cu have a positive binding energy, indicating that CO_2_ binds onto the Ru or Rh surface strongly while it does not bind to the other metal surfaces. This trend in CO_2_ binding energy on various metal catalyst agrees well with CO_2_ chemisorption results shown in Supplementary Table 5 and other previous report^36^. The strong adsorption of CO_2_ onto the Ru or Rh surface may change the electronic structure of the CO_2_. The electronic structure of free CO_2_ and CO_2_ adsorbed on the metal surface were calculated, and their HOMO (5*σ* bonding)–LUMO (2*π* anti-bonding) gaps are shown in Fig. 3b and Supplementary Fig. 11c. The gap decreased significantly from 8.5 eV for free CO_2_ to 2.4 eV for CO_2_ adsorbed on the Ru surface. The energy gap of the CO_2_ adsorbed on the Rh surface was 2.8 eV. These gaps are small enough for the electrons to jump under visible light irradiation. The gap was much larger as 4.5 eV for Ni, 3.8 eV for Pt, and 3.5 eV for Cu. In Fig. 2c and d, Ru had the absorption peak in the presence of CO_2_ at 528 nm (~2.3 eV) and Rh had the peak at 410 nm (~3.0 eV). The calculated energy gaps of 2.4 eV for Ru and 2.8 eV for Rh are in good agreement with the experimentally obtained values.

The diffuse reflectance infrared Fourier transform (DRIFT) spectra for CO_2_ adsorbed on the Ru surface are shown in Fig. 3c. The spectra obtained with and without light were compared. Whereas no peak appeared up to 200 °C without light, two distinct peaks were observed at 1950 and 1800 cm^−1^ even at 100 °C with light. The peak at 1950 cm^−1^ resulted from CO linearly adsorbed on Ru, and the peak at 1800 cm^−1^ indicated that CO was adsorbed on Ru through a bridge mode^37,38^. Upon light irradiation, CO_2_ would be cleaved to CO_ad_ and O_ad_ on the Ru surface. Supplementary Fig. 13 shows the DRIFT spectra obtained when H_2_ was additionally flowed after the CO_2_ was adsorbed on the Ru surface. The two peaks appeared at 150 °C without light, indicating that H_2_ assists in CO_2_ dissociation. However, the peaks were much larger with light, confirming that light irradiation clearly promoted CO_2_ dissociation.

The effect of light on CO hydrogenation was also investigated. Supplementary Fig. 14a shows the CO conversion data with and without light. The CO conversion was slightly higher under light irradiation, but the differences in the conversion with and without light were rather small. Supplementary Fig. 14b shows the DRIFT spectra when CO was adsorbed on the Ru surface, and Supplementary Fig. 14c shows the DRIFT spectra when H_2_ was additionally flowed after adsorbing CO onto the Ru surface. The spectra were almost identical, regardless of the light irradiation. CO hydrogenation was hardly affected by light irradiation, indicating that the enhanced activity upon light irradiation for CO_2_ hydrogenation is related to the CO_2_ dissociation. When CO_2_ is strongly adsorbed on the Ru surface, hybrid orbitals are formed with a much shorter energy gap. If the light can easily excite the electrons in the bonding orbitals to anti-bonding orbitals, then CO_2_ can be dissociated to CO. Further hydrogenation would produce CH_4_.

Kinetic analysis of light-assisted CO_2_ hydrogenation was performed with various CO_2_ partial pressures and temperatures. Supplementary Fig. 15a shows the dependences of CO_2_ conversion rates on the partial pressure of CO_2_. The CO_2_ conversion rate increased as the CO_2_ partial pressure increased, and reached a plateau at 4 kPa of CO_2_. The CO_2_ conversion rate followed the identical saturation behaviors under light irradiation, reaching the plateau at the same CO_2_ partial pressure. It indicates that the light irradiation did not change CO_2_ coverage on Ru surface, but interfere with the adsorbed CO_2_ species. The temperature dependence of CO_2_ hydrogenation around 150 °C was investigated as shown in Supplementary Fig. 15b. Without light, the effective energy barrier (*E*_a_) of Ru catalyst was 70.4 kJ mol^−1^, which agrees well with previous studies^39,40^. However, the *E*_a_ decreased significantly to 36.2 kJ mol^−1^ with light irradiation. The incident light lowers the effective energy barrier on Ru catalyst for CO_2_ hydrogenation.

### Comparing energy usage for thermal vs. light-assisted reactions

**Fig. 4:**
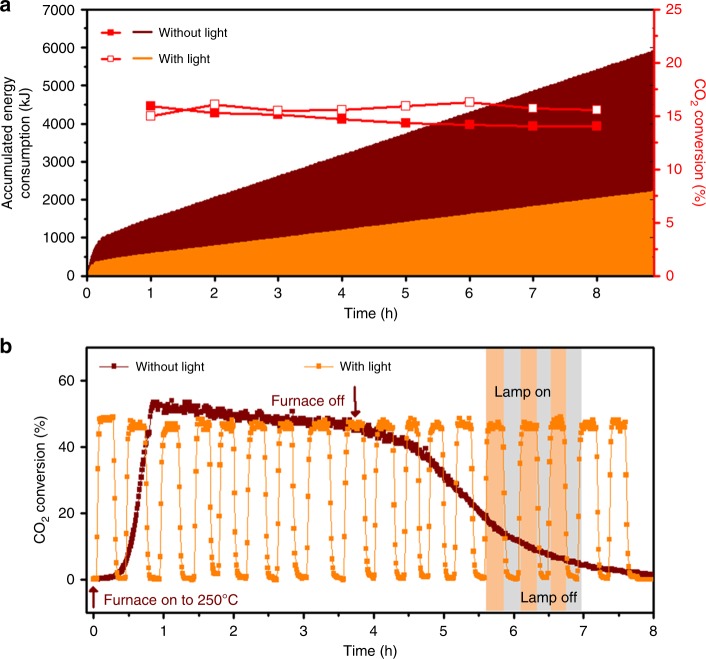
Energy usage and response for CO_2_ hydrogenation with or without light irradiation. **a** Energy consumption during CO_2_ hydrogenation with or without light irradiation. The condition in which both cases showed similar CO_2_ conversions was chosen; CO_2_ hydrogenation occurred at 180 °C without light, or 150 °C with light. 100 mW laser (532 nm) equipped with a beam expander was used as a light source. The overall power consumption was measured by a power meter. **b** Response test results for CO_2_ hydrogenation with or without light. At *t* = 0, the temperature of the reactor was set at 100 °C. In the case without light, the heater was turned on, the temperature was maintained for 3 h after it reached 250 °C, and the heater was turned off. In the case with light, a Xe lamp with a light intensity of 63 mW cm^−2^ was turned on for 15 min and turned off for 15 min repeatedly

The same CO_2_ conversion can be achieved at a lower temperature with light. If the power required to shine light is less than the power required to heat the reactor, the overall energy consumption would be reduced. Figure 4a shows the total energy consumption for CO_2_ hydrogenation with and without light. The reactor temperature was set to be 180 °C for the case without light, and 150 °C for the case with light. The CO_2_ conversion was maintained at ~15% for both cases. The total electric power consumed was measured by an online power meter. To minimize the energy consumption for shining a light, a compact laser with a wavelength of 532 nm was used with a beam expander instead of a Xe lamp. For a CO_2_ hydrogenation reaction for 8 h, the total energy consumption was 5411 kJ for the case without light, and 2020 kJ for the case with light. Only 37% of the energy was consumed when light was irradiated together. Clearly, the energy required for catalytic reactions in a continuous flow reactor can be significantly reduced by light irradiation.

In a thermally heated reactor, controlling the temperature instantaneously is nearly impossible. Figure 4b shows the response in CO_2_ conversion for turning on or off the heater or light source. When the heater was turned on at *t* = 0, it took 52 min to reach the target temperature of 250 °C and the highest conversion. More importantly, cooling down the reactor took much longer time. After the heater was turned off at *t* = 3.8 h, CO_2_ conversion was still observed with a long tail due to the residual heat in the reactor. However, the response became much faster with light irradiation. The highest conversion was immediately obtained after turning on the lamp. The CO_2_ conversion dropped to 0% within 3 min after turning off the lamp. The instantaneous control of CO_2_ conversion cannot be achieved with a conventional thermal reactor. This unique feature might be useful for on-demand CH_4_ production.

## Discussion

CO_2_ hydrogenation was performed on metallic catalysts of Ru, Rh, Pt, Ni, and Cu with and without light irradiation. The CO_2_ conversion was enhanced significantly on a Ru catalyst by light irradiation. The CO_2_ conversion was 1.6% at 150 °C without light on Ru, but the conversion increased to 32.6% at 150 °C with light. The effects of the Ru surface oxidation state and Ru nanoparticle size were evaluated. The light-enhancement appeared only at the metallic Ru surface, and larger nanoparticles exhibited more enhancement. Hot electrons generated upon light irradiation enhanced the CO_2_ conversion; however, the enhancement was not related to the light absorption on the metal surface. Rather, it was related to the HOMO-LUMO energy gap of the CO_2_ adsorbed on the metal surface. DFT calculations showed that CO_2_ is strongly bound to the Ru surface, and the energy gap is reduced from 8.5 eV in free CO_2_ to 2.4 eV in CO_2_ adsorbed on the Ru surface. Especially, light promotes CO_2_ dissociation to CO as confirmed by the DRIFTS data. Light irradiation could lower the reaction temperature for CO_2_ hydrogenation. When total energy consumption was compared at a CO_2_ conversion rate of 15%, the energy required for the case with light irradiation was only 37% of that for the case without light irradiation. Furthermore, the CO_2_ conversion could be instantaneously turned on and off by light irradiation unlike a conventional thermal reaction. This light-assisted heterogeneous reaction can contribute to lowering energy consumption in chemical production, with a much faster response for the changes in the reaction condition.

## Methods

### Catalyst synthesis

Metal nanoparticle catalysts supported on silica (M/SiO_2_, M = Pt, Ni, Cu, Ru, and Rh) were prepared using a wet impregnation method. Different amounts of metal precursors, (metal acetylacetonates; details in Supplementary Table 1) were dissolved in acetone (10 mL, Sigma-Aldrich) and added to silica nanopowder (1 g, Sigma-Aldrich). The slurry mixture was dried at 80 °C for 30 min, and it was placed into the furnace and heated up to 300 °C with a ramping rate of 10 °C min^-1^ in 10 vol% H_2_/N_2_ (200 sccm). Then, catalysts were reduced at 300 °C for 3 h. The extent of the surface oxidation on Ru/SiO_2_ was controlled by oxidizing the catalyst at 500 °C under static air for various treatment times (1 or 60 min).

### CO_2_ hydrogenation

The M/SiO_2_ (M = Pt, Ni, Cu, Ru, and Rh) catalysts were placed in a cylindrical quartz sample holder (2 cm of diameter). Metal 10 mg was loaded for all the reactions. The sample holder was placed in a customized photoreactor equipped with a sapphire glass window (a thickness of 23 mm) as shown in Supplementary Fig. 2. It should be noted that a water circulating filter was located in front of the Xe lamp (300 W, Newport) to exclude the effect of thermal radiation from the lamp. The catalysts were reduced prior to the CO_2_ hydrogenation for 3 h at 300 °C under 10 vol% H_2_/N_2_ flow. In a typical CO_2_ hydrogenation, a mixture of 0.5 vol% CO_2_/N_2_ (50 sccm) and H_2_ (1.5 sccm) was fed into the reactor through a static mixer with or without light irradiation at atmospheric pressure. The CO_2_ conversion was monitored by an online gas chromatograph (YL6100 GC, YL Instrument) equipped with molesieve/PORAPAK N columns (Sigma-Aldrich), a thermal conductivity detector (for H_2_ and N_2_ detection), and a flame ionization detector (for CH_4_, CO, and CO_2_ detection) with a methanizer. Quantitative analysis was performed using N_2_ as an internal standard. The dependency on the light intensity or light wavelength was measured at 150 °C for Ru and 200 °C for Rh. The light intensity was varied by changing the electric power of the lamp and measured by a spectroradiometer (CS2000, KONICA MINOLTA). The transmittances of the liquid filter and the sapphire glass window are shown in Supplementary Fig. 16a,b. The light intensity was corrected excluding the effect of the liquid filter and the sapphire glass window. The photoaction spectra were obtained using a monochromator (MonoRa151i, Dongwoo Optron) with wavelengths shown in Supplementary Fig. 16c. A 532 nm laser (100 mW, CNI laser) was used as the light source for the energy consumption test.

### Estimation of the QY

The energy of the irradiated light for a given time is:

1 $${{E}} = {{n}}\frac{{hc}}{\lambda } = IAs,$$

where *n* is a number of incident photon, *λ* is a wavelength of incident photon, *h* is Planck constant, *c* is a speed of light, *I* is the light intensity, *A* is the area of irradiation, and *s* is irradiation time. Then incident photon flux is defined as:

2 $$\frac{n}{s} = \frac{E}{s}\frac{\lambda }{{hc}} = IA\frac{\lambda }{{hc}}.$$

The QY can be estimated as:

3 $${\mathrm{QY}} = \frac{{n_{{\mathrm{CO}}_2}/s}}{{n/s}},$$

where $n_{{\mathrm{CO}}_2}$ is the number of the converted CO_2_ molecules. The wavelength-dependent measurement was performed using a monochromator (MonoRa151i, Dongwoo Optron). In prior to the measurement, the monochromatic light was calibrated with a spectrometer (Maya 2000 Pro, Ocean Optics) as shown in Supplementary Fig. 16c. The intensity of the monochromatic light was measured with an optical power meter (PM204, Thorlab) after the monochromatic light passed through a sapphire window of the reactor. The catalytic activity under the monochromatic light irradiation was measured at steady state for CO_2_ hydrogenation. The rate of the CO_2_ molecules converted by the incident photon was estimated excluding the CO_2_ conversion from thermal reaction.

### Characterizations

The morphology of prepared catalysts was observed using HR-TEM (TECNAI). A powder X-ray diffractometer (SmartLab, RIGAKU) was used to determine the crystalline size of the metal nanoparticles. X-ray photoelectron spectroscopy (XPS, Thermo VG Scientific) equipped with a monochromatic Al Kα X-ray source was used to measure the surface properties of metal nanoparticles. The binding energies were calibrated with adventitious C 1 s signal at 285 eV as a reference. The absorption spectra of the metal catalysts were obtained using an UV-diffuse reflectance spectrometer (UV-DRS) (UV3600, Shimadzu). Elemental analysis was conducted using an inductively coupled plasma optical emission spectrometer (ICP-OES 720, Agilent) to confirm the actual metal content in the M/SiO_2_ catalysts. In situ diffuse reflectance infrared Fourier transform spectroscopy (DRIFTS) measurements were performed using a FT-IR spectrometer (Thermo, Nicolet 6700) equipped with a MCT detector. In situ UV–Vis spectroscopy was performed using a UV–Vis spectrophotometer (Perkin Elmer, lambda 1050) equipped with diffuse reflectance accessory (PIKE, DiffuseIR) enabling gas flow and temperature control. The power consumption during the CO_2_ hydrogenation was monitored using a 2-channel power meter (WT500, YOKOGAWA) which contacted both furnace and light sources. The energy consumption by the standby power was excluded for both cases.

### Theoretical calculation using DFT

All of the quantum chemical calculations were performed with the Vienna ab initio simulation package^41^. We used the projector augmented wave pseudopotentials with Perdew–Burke Ernzerhof^42^ exchange correlation functional. The energy cutoff was set as 500 eV, while a 5 × 5 × 1 *k*-point grid was used for energy relaxation, and 20 × 20 × 1 for the density of states calculation using the Monkhorst–Pack scheme^43^. Five metal catalysts (Cu, Ni, Pt, Rh, and Ru) were modeled using four layered slabs with the surfaces of (111) used for Cu, Ni, Pt, and Rh, and (0001) for Ru with a 15 Å vacuum region. Only the atoms from the top layer could relax during the DFT simulations. The CO_2_ molecules were initialized on different atomic sites (Supplementary Fig. 11), and the binding energy was determined by the lowest energy configurations (Supplementary Fig. 12). The angles of the CO_2_ molecules are slightly bent due to the strong interaction between the metal surfaces and the CO_2_ molecule. The CO_2_ binding energy was defined as follows:

4 $$E_{{\mathrm{bind}}} = E_{{\mathrm{catalyst}} + {\mathrm{CO}}_2} - (E_{{\mathrm{catalyst}}} + E_{{\mathrm{CO}}_2}),$$

where $E_{{\mathrm{bind}}}$ is the binding energy of CO_2_ on the metal catalyst, and $E_{{\mathrm{catalyst}} + {\mathrm{CO}}_2}$ is the total energy of the metal catalyst with a single CO_2_ molecule. $E_{{\mathrm{catalyst}}}$ and $E_{{\mathrm{CO}}_2}$ are the total energy of free metal catalyst and CO_2_ molecule, respectively.

### Data availability

The authors declare that the data supporting the findings of this study are available within the paper and its Supplementary Information files.

## Electronic supplementary material

Supplementary Information
