# Supplementary material for: Energy-efficient CO2 hydrogenation with fast response using photoexcitation of CO2 adsorbed on metal catalysts
Source: Nat Commun. 2018 Aug 2;9:3027. doi: 10.1038/s41467-018-05542-5 (PMC6072744; doi:10.1038/s41467-018-05542-5)
Supplement: Supplementary file 1 — Supplementary Information [file 41467_2018_5542_MOESM1_ESM.pdf]

Supplementary Information

Energy-Efficient CO_2 Hydrogenation with Fast Response Using Photoexcitation of CO_2 Adsorbed on Metal Catalysts

Lee et al.

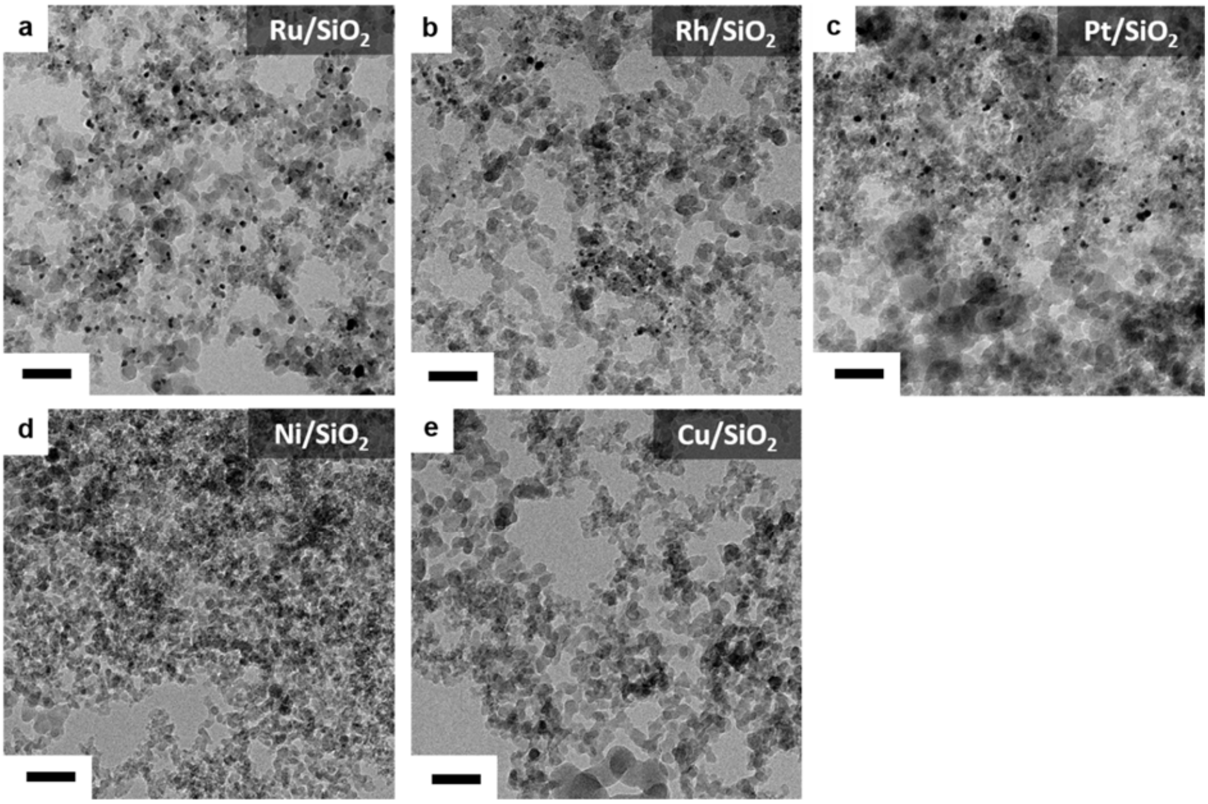

Supplementary Figure 1. TEM images of typical catalysts

(a) Ru/SiO_2, (b) Rh/SiO_2, (c) Pt/SiO_2, (d) Ni/SiO_2 and (e) Cu/SiO_2 catalysts. The scale bar is 50 nm.

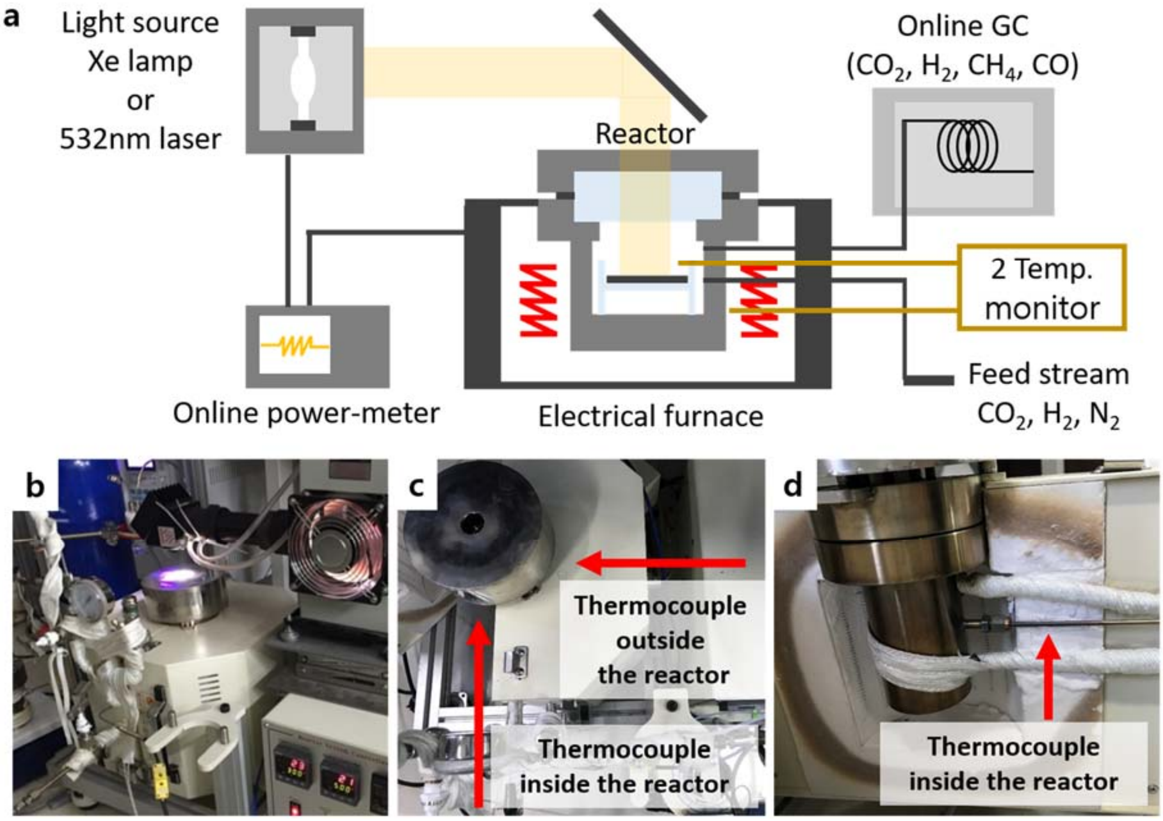

Supplementary Figure 2. Customized photo-reactor system for light-assisted CO_2 hydrogenation

(a) a scheme and (b-d) photographs.

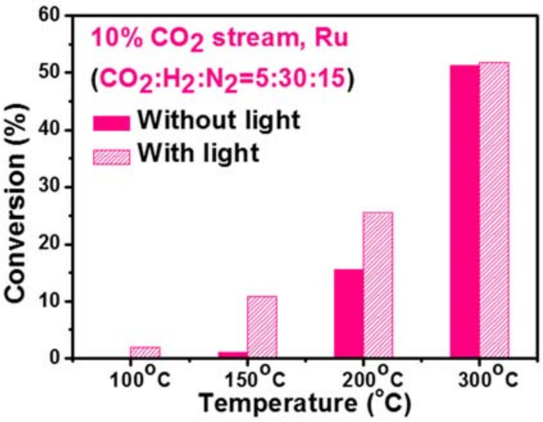

Supplementary Figure 3. CO_2 hydrogenation when a high concentration of CO_2 was used

Light-assisted hydrogenation of high concentration CO_2 (10 %) on Ru/SiO_2 catalysts with a Ru size of 5.6 nm. 10 mg of Ru was used for the reaction. A mixture of CO_2/H_2/N_2 (5/30/15 sccm) was used as a feed stream. 63 mW/cm^2 of Xe lamp was used as a light source.

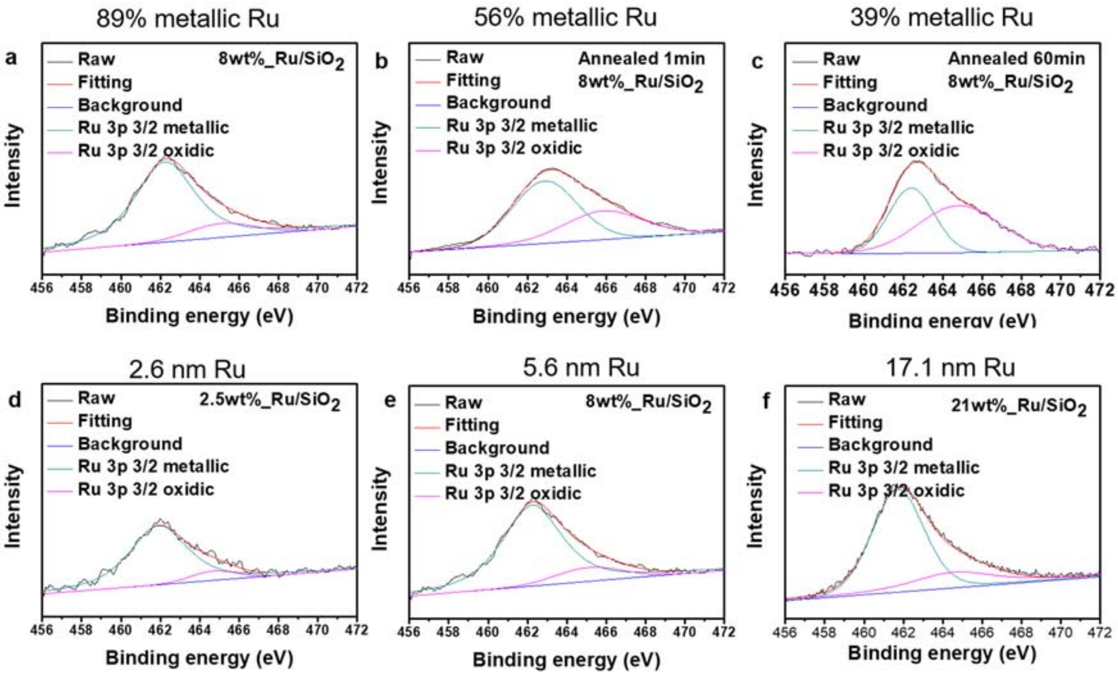

Supplementary Figure 4. XPS data for the Ru catalysts
with different Ru oxidation states; (a) 89% metallic Ru, (b) 56% metallic Ru, (c) 39% metallic Ru, and different Ru sizes; (d) 2.6 nm Ru, (e) 5.6 nm Ru, (f) 17.1 nm Ru.

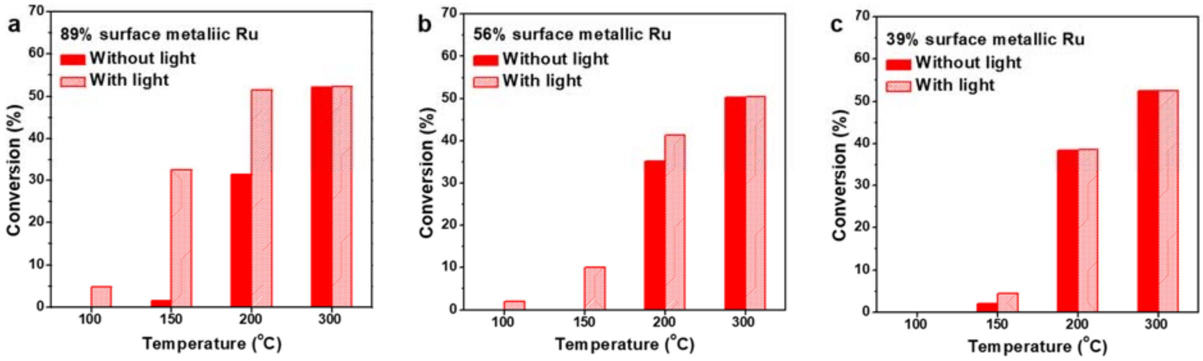

Supplementary Figure 5. Effect of surface Ru oxide on CO_2 conversion for CO_2 hydrogenation

Ru/SiO_2 with a Ru size of 5.6 nm were annealed at 500 °C in air for 1 min or 60 min. The as-made sample contained 89% of metallic Ru at the surface, while the annealed sample for 1 min contained 56% of metallic Ru and the annealed sample for 60 min contained 39% of metallic Ru. The percentages of metallic Ru were estimated from XPS data.

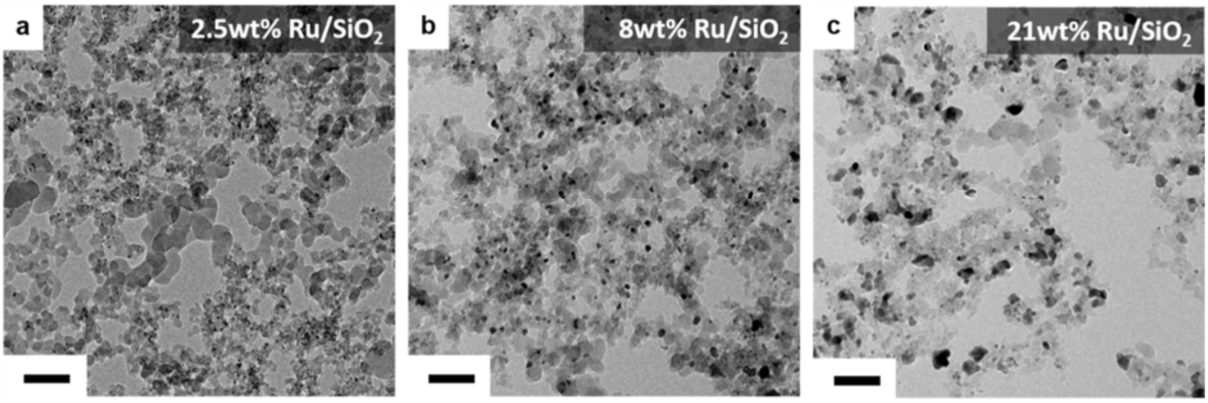

Supplementary Figure 6. TEM images for Ru/SiO_2 with different Ru loading amounts
(a) 2.5 wt% Ru/SiO_2, (b) 8 wt% Ru/SiO_2, and (c) 21 wt% Ru/SiO_2. The scale bar is 50 nm.

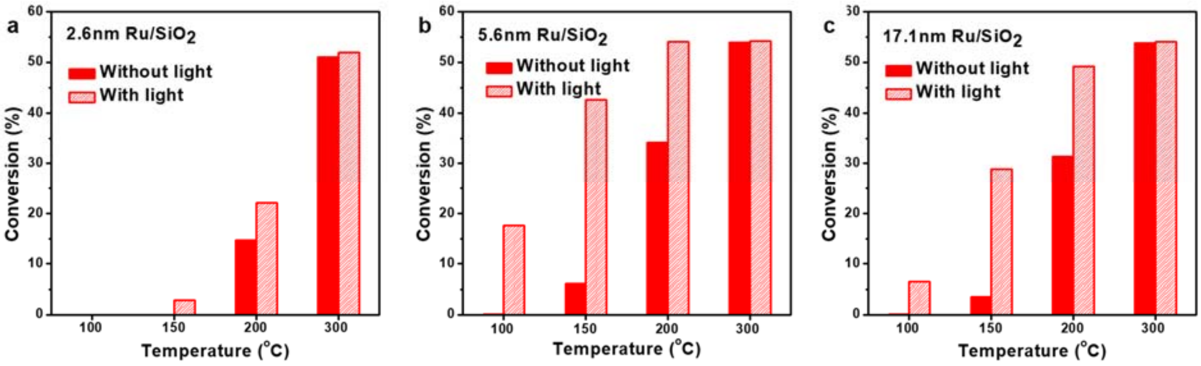

Supplementary Figure 7. CO_2 conversion for CO_2 hydrogenation on Ru/SiO_2 with different Ru sizes

As the Ru content in Ru/SiO_2 increased from 2.5 wt% to 8 wt% to 21 wt%, the Ru domain size increased from 2.6 nm to 5.6 nm to 17.1 nm.

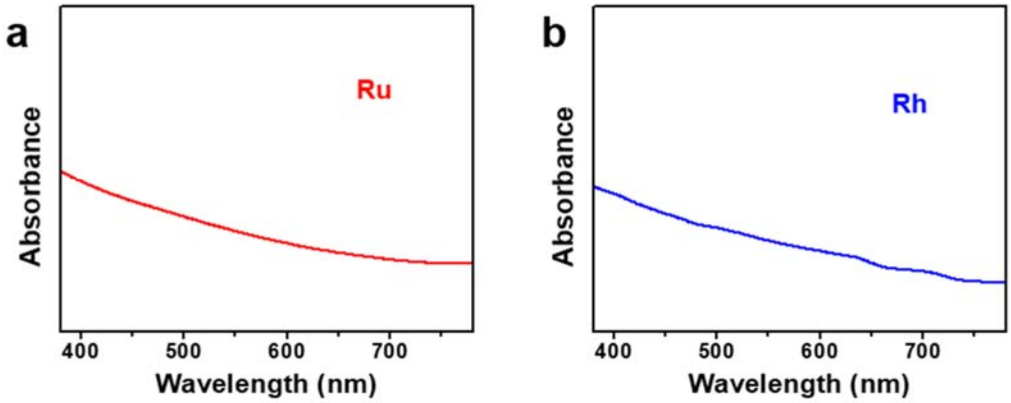

Supplementary Figure 8. UV-DRS spectra obtained without CO_2 flow
(a) Rh/SiO_2 and (b) Rh/SiO_2.

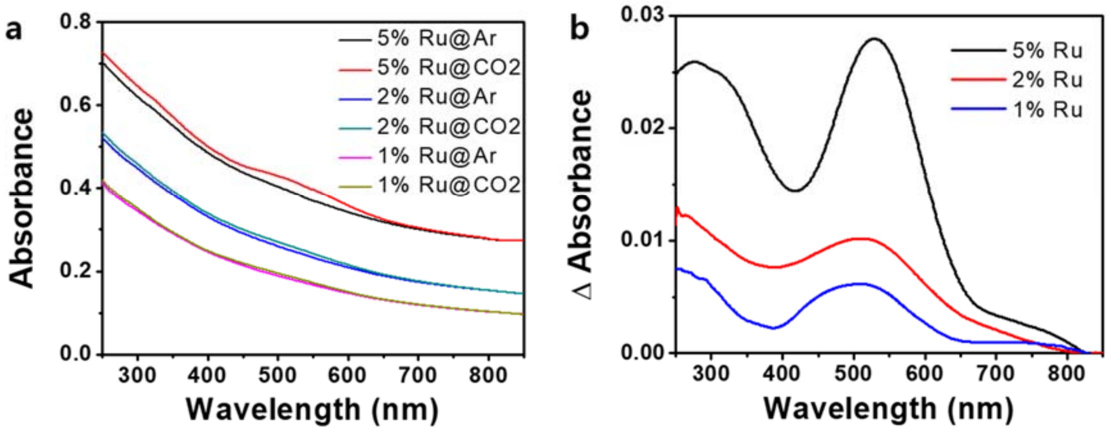

Supplementary Figure 9. In-situ UV-Vis spectroscopy using Ru/SiO_2 catalysts in Ar or CO_2 flow

(a) raw data and (b) the difference between Ar flow and CO_2 flow. The samples were diluted with KBr; the weight percentage of Ru/SiO_2 was 1, 2, and 5 %. Δ Absorbance was calculated by subtracting the absorbance in Ar flow (100 sccm) from the absorbance in CO_2 flow (100 sccm).

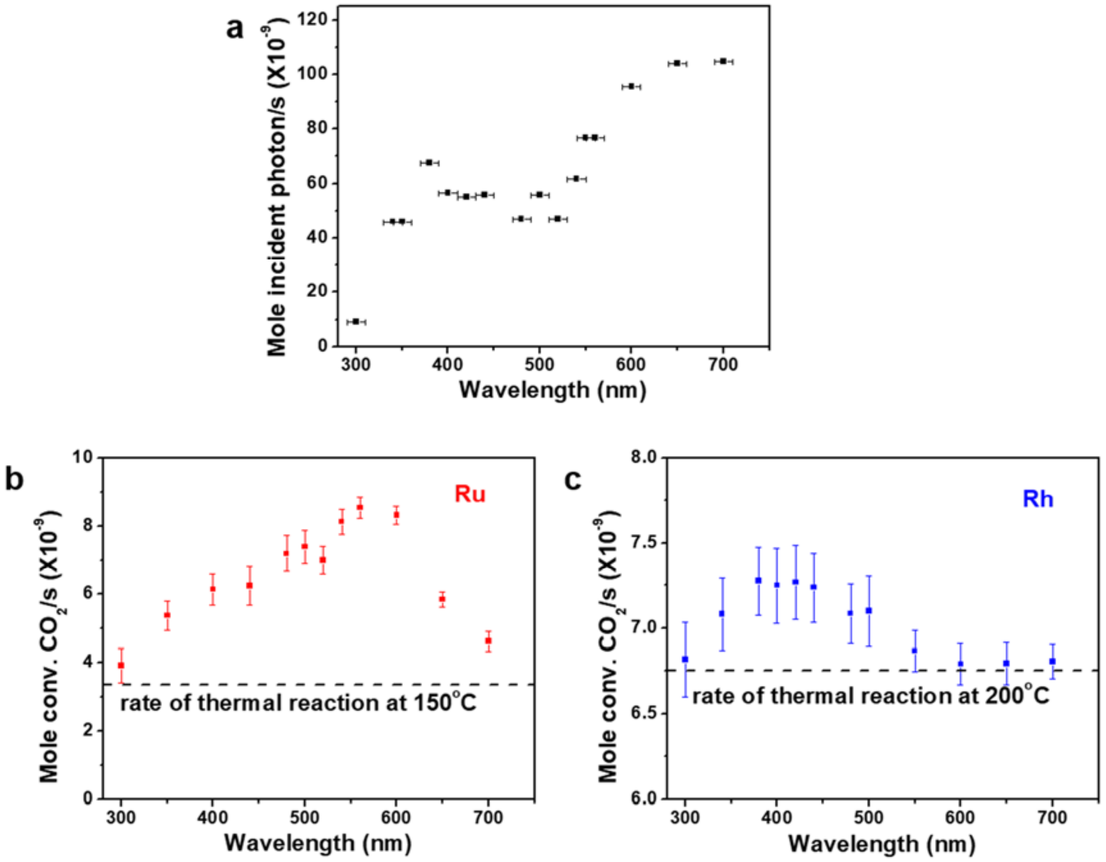

Supplementary Figure 10. Estimation of quantum yields using monochromic light
(a) The molar flow rate of incident photons at each wavelength. Error bars in x-direction indicates full-width at half maximum intensity of monochromatic light. (b, c) The molar rate of CO_2 converted on Ru or Rh at each wavelength. Error bars in y-direction indicates a deviation among three different measurements.

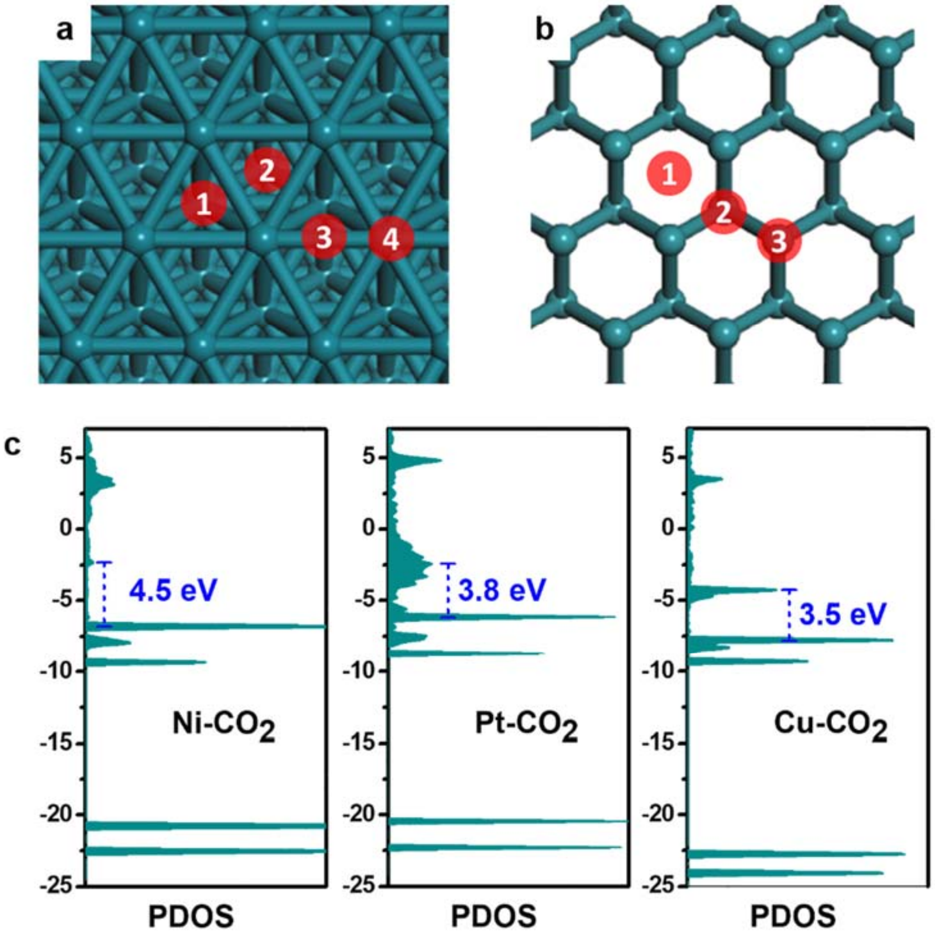

Supplementary Figure 11. Modeled metal surface structure and electronic structure of the adsorbed CO_2 on Ni, Pt, and Cu

(a) Top view of (111) surface for fcc structure (Cu, Pt, Ni, Rh). The labeled sites are ① fcc hollow, ② hcp hollow, ③ bridge, and ④ on top. (b) Top view of (0001) surface for hcp structure (Ru). The labeled sites are ① hcp hollow, ② fcc hollow, and ③ on top. The initial configurations of CO_2 molecule on the metal surface were constructed with different orientations on these labeled sites. (c) The electronic structure of adsorbed CO_2 over Ni, Pt, and Cu surfaces was calculated using DFT.

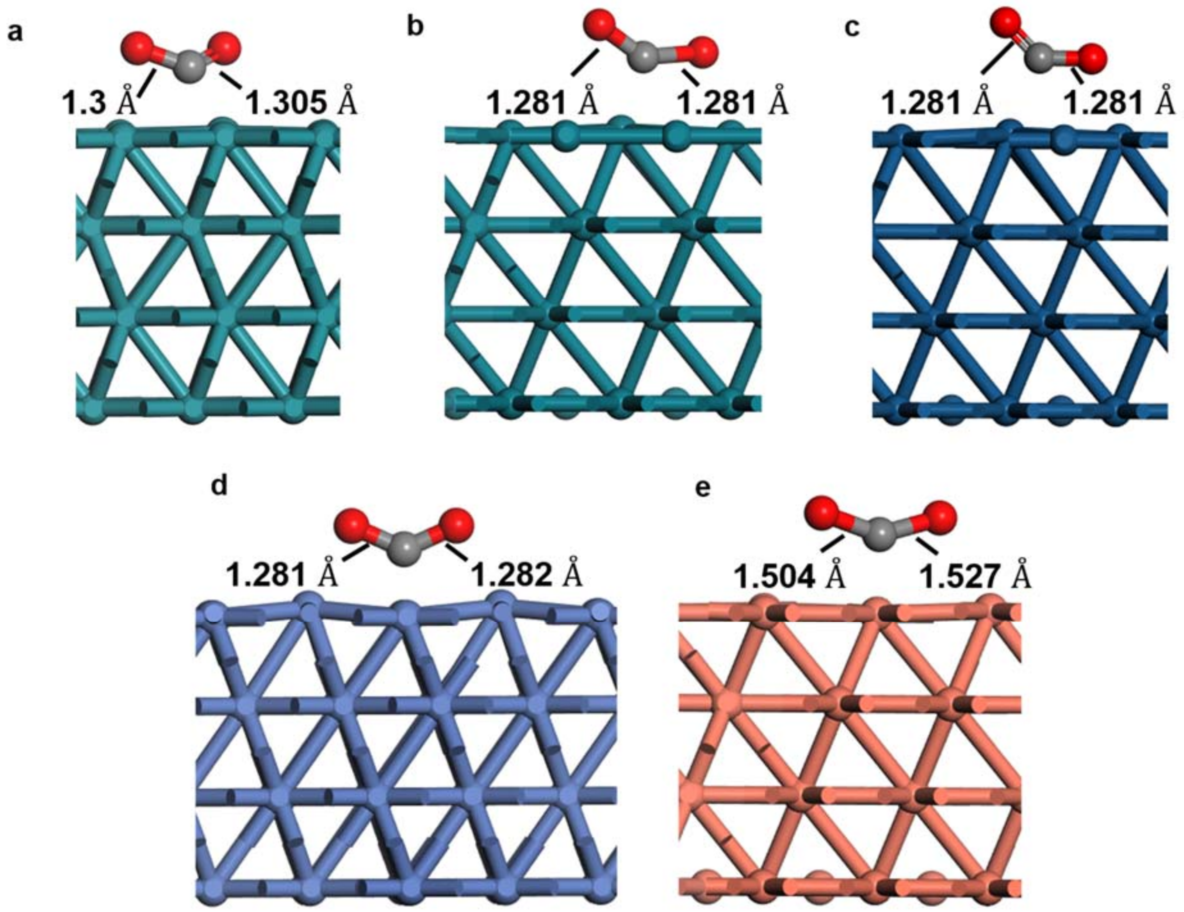

Supplementary Figure 12. Optimized CO_2 molecules on the various catalysts
(a) Ru, (b) Rh, (c) Pt, (d) Ni and (e) Cu.

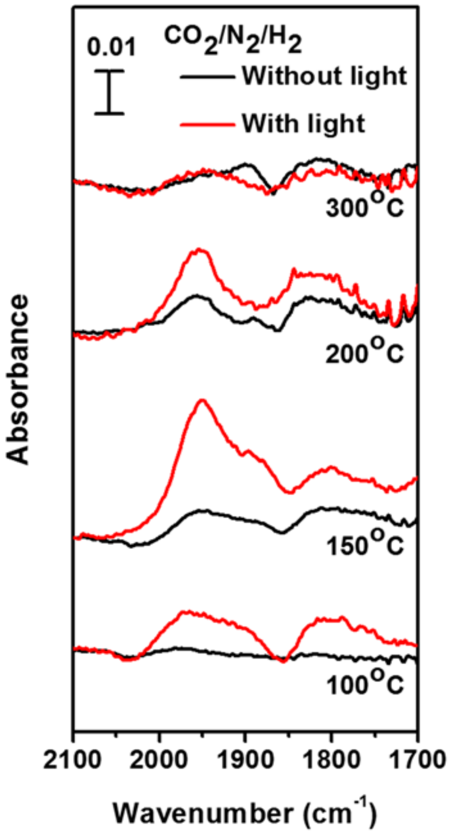

Supplementary Figure 13. DRIFT spectra for hydrogenation of absorbed CO_2 over Ru catalyst

0.5% CO_2/N_2 was flown at 50 sccm for 10 min, the cell was purged with N_2 at 50 sccm for 15 min to desorb the weakly adsorbed CO_2, diluted hydrogen stream (H_2 at 1.5 sccm and N_2 at 50 sccm) was provided for 10 min, then the IR spectra were obtained.

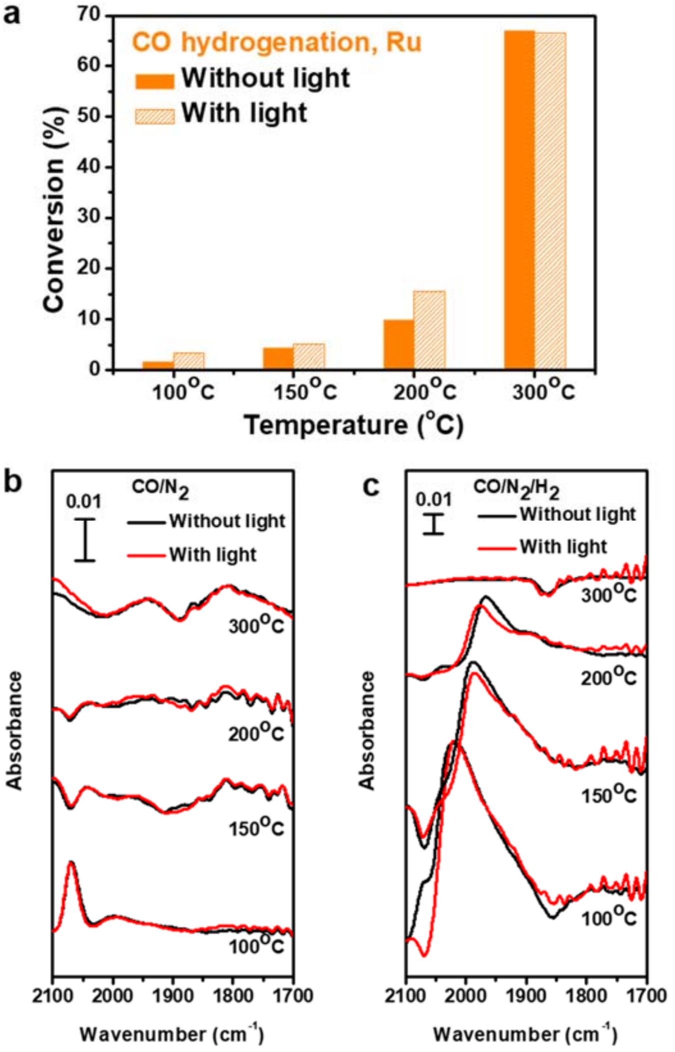

Supplementary Figure 14. CO hydrogenation with or without light irradiation

(a) The effect of light on CO hydrogenation on Ru/SiO_2 catalyst with a Ru size of 5.6 nm. DRIFT spectra for (b) CO adsorption and (c) hydrogenation of absorbed CO over Ru catalyst. 1% CO/N_2 was flown at 50 sccm for 10 min, the cell was purged with N_2 at 50 sccm for 15 min to desorb the weakly adsorbed CO. In a case of (b), the IR spectra were obtained after N_2 purging. The spectra in (c) were obtained after flowing diluted hydrogen (1.5 sccm of H_2 + 50 sccm of N_2) for 10 min.

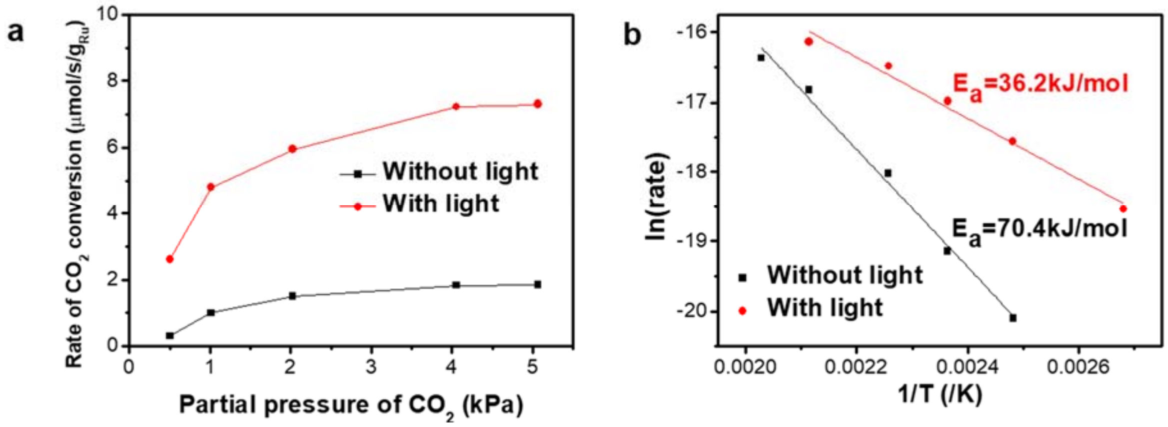

Supplementary Figure 15. Dependence on CO_2 partial pressure and temperature
(a) The dependence on CO_2 partial pressure at 150˚C and (b) the dependence on temperature for CO_2 hydrogenation using Ru catalyst with and without light. The partial pressure of H_2 in (a) was 20 kPa. The partial pressures of CO_2 and H_2 in (b) were 5 kPa and 20 kPa, respectively. The total pressure was 101.325 kPa.

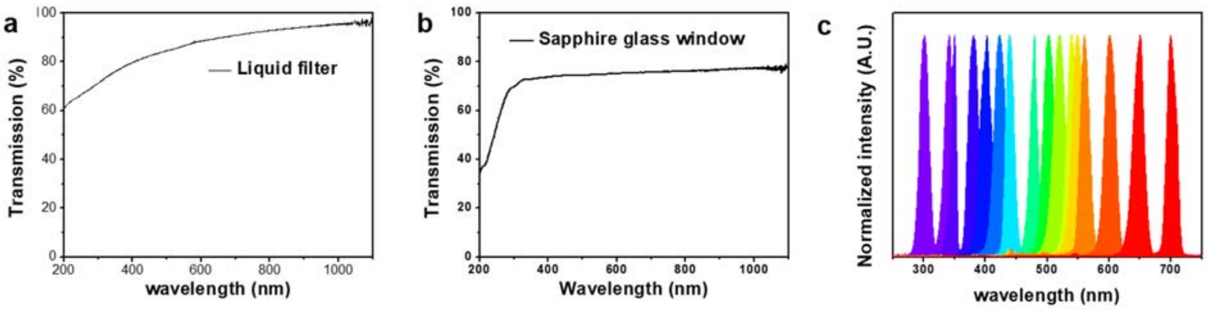

Supplementary Figure 16. Transmittance and monochromic light

Transmittance of the (a) liquid filter, which consists of fused silica window, and the (b) sapphire glass window in photo-reactor. (c) Monochromatic light with various wavelengths used for obtaining the photoaction spectra.

Supplementary Table 1. Preparation conditions for various metal catalysts. All the samples were reduced at 300 °C (ramping rate: 10˚C min^{-1}) for 3 h using 10% H_2/N_2 flow.

	Metal Precursors[a]	Metal weight %	Metal crystalline size (nm)[b]
Ru/SiO_2	$Ru(acac)_3$	2.5	2.6
		8	5.6
		21	17.1
Rh/SiO_2	$Rh(acac)_3$	1	6.1
		2	7.1
		5	10.6
		10	17.0
Pt/SiO_2	$Pt(acac)_2$	0.5	6.6
		1	6.9
		5	10.6
		10	15.6
Cu/SiO_2	$Cu(acac)_2$	1	3.9
		2	7.2
		5	22.5
		10	30.6
Ni/SiO_2	$Ni(acac)_2$	1	4.0
		2	7.4
		3	9.8

[a] 'acac' indicates acetylacetonate; [b] The size was determined using a Scherrer equation from powder XRD data. The samples shaded with grey in each metal catalyst were used in Figure 1.

Supplementary Table 2. Full reaction data of CO_2 hydrogenation on Ru, Rh, Pt, Ni and Cu catalysts

		Temperature (°C)											
		100			150			200			300		
		CO_2 Conversion (%) / Rate of consumption [μmol hr^{-1}·g$_{metal}^{-1}$]	Selectivity (%) / Rate of production [μmol hr^{-1}·g$_{metal}^{-1}$]		CO_2 Conversion (%) / Rate of consumption [μmol hr^{-1}·g$_{metal}^{-1}$]	Selectivity (%) / Rate of production [μmol hr^{-1}·g$_{metal}^{-1}$]		CO_2 Conversion (%) / Rate of consumption [μmol hr^{-1}·g$_{metal}^{-1}$]	Selectivity (%) / Rate of production [μmol hr^{-1}·g$_{metal}^{-1}$]		CO_2 Conversion (%) / Rate of consumption [μmol hr^{-1}·g$_{metal}^{-1}$]	Selectivity (%) / Rate of production [μmol hr^{-1}·g$_{metal}^{-1}$]	
			CH_4	CO		CH_4	CO		CH_4	CO		CH_4	CO
Ru 5.6nm 89% metallic	Without light	0	-	-	1.6<br>±0.3<br>[1071]	99.9<br>±0.1<br>[1070]	0	28.4<br>±0.9<br>[19018]	99.9<br>±0.1<br>[19002]	0	52.1<br>±1.3<br>[34888]	99.9<br>±0.1<br>[34859]	0
	With light	4.7<br>±0.2<br>[3147]	99.9<br>±0.1<br>[3144]	0	32.6<br>±0.7<br>[21830]	99.9<br>±0.1<br>[21810]	0	51.5<br>±0.9<br>[34487]	99.9<br>±0.1<br>[34459]	0	52.4<br>±0.7<br>[35089]	99.9<br>±0.1<br>[35054]	0
Ru 5.6nm 56% metallic	Without light	0			0	-	-	35.4<br>±0.7<br>[23705]	99.9<br>±0.1<br>[23682]	0	50.1<br>±0.2<br>[33549]	99.9<br>±0.1<br>[33516]	0
	With light	2<br>±0.1<br>[1339]	99.9<br>±0.1<br>[1338]	0	10.1<br>±0.1<br>[6763]	99.9<br>±0.1<br>[6757]	0	41.2<br>±0.2<br>[27589]	99.9<br>±0.1<br>[27566]	0	50.4<br>±0.4<br>[33750]	99.9<br>±0.1<br>[33716]	0
Ru 5.6nm 39% metallic	Without light	0	-	-	1.9<br>±0.2<br>[1272]	99.9<br>±0.1<br>[1270]	0	38.2<br>±0.2<br>[25580]	99.9<br>±0.1<br>[25555]	0	52.4<br>±0.1<br>[35089]	99.9<br>±0.1<br>[35054]	0
	With light	0	-	-	4.4<br>±0.2<br>[2946]	99.9<br>±0.1<br>[2943]	0	38.6<br>±0.3<br>[25848]	99.9<br>±0.1<br>[25822]	0	52.5<br>±0.2<br>[35156]	99.9<br>±0.1<br>[35122]	0
Ru 2.6nm 91% metallic	Without light	0	-	-	0	-	-	14.5<br>±0.2<br>[9709]	99.9<br>±0.1<br>[9701]	0	50.6<br>±0.3<br>[33884]	99.9<br>±0.1<br>[33855]	0
	With light	0	-	-	2.8<br>±0.1<br>[1875]	99.9<br>±0.1<br>[1873]	0	21.9<br>±0.2<br>[14665]	99.9<br>±0.1<br>[14651]	0	51.3<br>±0.4<br>[34352]	99.9<br>±0.1<br>[34318]	0
Ru 17.1nm 89% metallic	Without light	0	-	-	3.5<br>±0.4<br>[2343]	99.9<br>±0.1<br>[2341]	0	31.9<br>±0.3<br>[21362]	99.9<br>±0.1<br>[21344]	0	53.3<br>±0.3<br>[35692]	99.9<br>±0.1<br>[35666]	0
	With light	6.1<br>±0.2<br>[4085]	99.9<br>±0.1<br>[4083]	0	29.1<br>±0.3<br>[19487]	99.9<br>±0.1<br>[19467]	0	49.2<br>±0.3<br>[32946]	99.9<br>±0.1<br>[32914]	0	53.1<br>±0.7<br>[35558]	99.9<br>±0.1<br>[35552]	0
Pt	Without light	0	-	-	0	-	-	0	-	-	8.1<br>±1.0<br>[5424]	0	99.9<br>±0.1<br>[5420]
	With light	0	-	-	0	-	-	0	-	-	8.1<br>±0.8<br>[5424]	0	99.9<br>±0.1<br>[5420]
Ni	Without light	0	-	-	0	-	-	0	-	-	2.8<br>±0.3<br>[1875]	10.9<br>±0.1<br>[205]	89<br>±0.1<br>[1669]
	With light	0	-	-	0	-	-	0	-	-	2.8<br>±0.3<br>[1876]	10.9<br>±0.1<br>[205]	89<br>±0.1<br>[1670]
Cu	Without light	0	-	-	0	-	-	0	-	-	1.9<br>±0.3<br>[1272]	0	99.9<br>±0.2<br>[1270]
	With light	0	-	-	0	-	-	0	-	-	1.9<br>±0.4<br>[1272	0	99.9<br>±0.2<br>[1271]

		Temperature (°C)											
		150			200			250			300		
Rh	Without light	0	-	-	7.4<br>±0.3<br>[4955]	99.9<br>±0.1<br>[4950]	0	9.4<br>±0.4<br>[6295]	99.9<br>±0.1<br>[6288]	0	11.5<br>±0.4<br>[7701]	99.9<br>±0.1<br>[7699]	0
	With light	0	-	-	11.5<br>±0.4<br>[7701]	99.9<br>±0.1<br>[7693]	0	13.3<br>±0.4<br>[8906]	99.9<br>±0.1<br>[8899]	0	16.5<br>±0.5<br>[11049]	99.9<br>±0.1<br>[11039]	0

Supplementary Table 3. Comparison of the production rates with literature values for gas-phase CO_2 hydrogenation under light irradiation.

Catalysts	Temp (˚C)	Light source	Light intensity	Conv. (%)	Production rate (μmol hr^{-1}·g$_{metal}^{-1}$)		Note	Ref.
					CH_4	CO		
8 % Ru/SiO_2	100	Xe	63 mW cm^{-2}	1.9	25446	-	Flow reactor (inlet 10% CO_2)	This work
	150			10.9	145982	-		
	200			25.5	341517	-		
	300			51.8	693750	-		
3.8 % Ru/RuO_2 /TiO_2	46	Solar simulator	80 mW cm^{-2}	98	51.8	-	Batch reactor	1
Ru/Si (nanowires)	150	Xe	320 mW cm^{-2}	-	990	-	Batch reactor	2
NiO(1 %)-In_2O_3 (3.5 %)/TiO_2	120	-	150 mW cm^{-2}	10.2	34	12029	Batch reactor	3
Ni/$SiO_2Al_2O_3$	150	Solar simulator	-	94.9	54608	1573	Batch reactor	4
NiO				89.8	13290	-		
Fe_2O_3				51	325	7171		
CoO				27.4	1569	2453		
2.4 % Ru/Al_2O_3	370	Xe	300 W	95.8	300000	-	Photo-thermal, Batch reactor	5
2.6 % Rh/Al_2O_3	370			96.3	156000	-		
2.1 % Ni/Al_2O_3	380			93.3	48000	3000		
2.5 % Co/Al_2O_3	350			92.6	21000	6000		
2.0 % Pd/Al_2O_3	370			93.4	8000	2000		
2.4 % Pt/Al_2O_3	350			60.4	600	9600		
2.8 % Ir/Al_2O_3	320			14.9	720	640		
2.4 % Fe/Al_2O_3	320			7.3	30	420		

Supplementary Table 4. Characterization results of Ru/SiO_2 catalysts with different Ru sizes. The metallic state of Ru surface on each catalyst was controlled to the similar level by reducing the catalyst under 200 sccm of 10 % H_2/N_2 flow at 300 °C.

Ru wt%	Ru size[a] (nm)	H_2 uptake (mmol g_{Ru}^{-1})	Ru size[b] (nm)	Metallic Ru[c] (%)
2.5	2.6	3.42	3.8	91.3
8	5.6	0.95	7.2	90.1
21	17.1	0.12	20.1	89.2

[a] Ru size was estimated from powder XRD data; [b] Ru size was estimated from H_2 uptakes data; [c] The percentages of metallic Ru on the surface was estimated from XPS data.

Supplementary Table 5. CO_2 chemisorption results for various metal catalysts supported on silica.[a]

	Ru	Rh	Ni	Pt	Cu
CO_2 uptakes (μmol g_{metal}^{-1})	40.3	18.5	3.9	1.9	0.4

[a]The CO_2 chemisorption experiment was conducted using BELCAT-B (BEL, Japan). All the catalysts were pre-treated by 5% H_2/Ar (50 sccm) at 300 °C for 1 h. Subsequently, the gas flow was switched to pure He (50 sccm) and the catalysts were kept for 1 h at 300 °C, then cooled to room temperature. CO_2 chemisorption was performed using a pulse injection method with 5% CO_2/He gas at 40 °C.

Supplementary References

1. Thampi, K. R., *et al.* Methanation and photo-methanation of carbon dioxide at room temperature and atmospheric pressure. *Nature* **327**, 506 (1987).
2. O'Brien, P. G., *et al.* Photomethanation of Gaseous CO_2 over Ru/Silicon Nanowire Catalysts with Visible and Near-Infrared Photons. *Adv. Sci.* **1**, 1400001 (2014).
3. Tahir, M., *et al.* Performance analysis of nanostructured NiO–In_2O_3/TiO_2 catalyst for CO_2 photoreduction with H_2 in a monolith photoreactor. *Chem. Eng. J.* **285**, 635-649 (2016).
4. Sastre, F., *et al.* Complete Photocatalytic Reduction of CO_2 to Methane by H_2 under Solar Light Irradiation. *J. Am. Chem. Soc.* **136**, 6798-6801 (2014).
5. Meng, X., *et al.* Photothermal Conversion of CO_2 into CH_4 with H_2 over Group VIII Nanocatalysts: An Alternative Approach for Solar Fuel Production. *Angew. Chem. Int. Ed.* **53**, 11478-11482 (2014).
